# Treatment of lymph node metastases from gastric cancer with a combination of Irreversible Electroporation and Electrochemotherapy: a case report

**DOI:** 10.1002/ccr3.1079

**Published:** 2017-07-14

**Authors:** Nina Klein, Stefan Zapf, Enric Gunther, Michael Stehling

**Affiliations:** ^1^ Tumortherapie Center Institut für Bildgebende Diagnostik Strahlenbergerstr. 110 63067 Offenbach am Main Germany

**Keywords:** Electrochemotherapy, focal therapy, gastric cancer, Irreversible Electroporation, lymph node metastases

## Abstract

The combination of Irreversible Electroporation and Electrochemotherapy (IRECT) was well tolerated, safe, and had antitumor activity in this case study of a patient with lymph node metastases from gastric cancer. We therefore recommend the consideration of further clinical studies to investigate the treatment of cancerous tissue with IRECT.

## Background

Gastric cancer is a common form of malignant disease: Worldwide, it ranks second in all causes of death caused by cancer 1. A formidable percentage of patients are considered incurable at diagnosis due to disseminated disease 2, and there is a high recurrence rate even in patients with resectable tumors 3. Gastrectomy is often accompanied by a dissection of the lymph nodes of group 1 (perigastric) or group 2 (primary vessels to the stomach) lymph nodes, but lymphadenectomy is not always required, as not all patients have lymph node metastases. In cases of lymph node metastases occurrence, a gentle, minimally‐invasive alternative for the treatment of lymph node metastases could be beneficial.

Electrochemotherapy (ECT) is a focal treatment of cancerous tissue which combines the effect of reversible cell electroporation with pharmaceutical agents to ablate malignant cells locally in solid cancers. Electroporation can be achieved by applying short high‐intensity pulsed electric fields to cells, causing transient partial permeability of cell membranes due to the induction of a large transmembrane voltage. The pores which get transiently formed in the process will in turn cause a significant increase in cellular uptake of cytotoxic agents such as bleomycin, and therefore increasing their cytotoxicity 4, 5, 6, 7. Bleomycin is a cytotoxic molecule which, under normal circumstances, does not cross the cell membrane easily. As it disrupts cellular mitosis, it is more effective in malignant cells, which have a higher mitotic rate than healthy cells, resulting in a partial tumor cell selectivity of ECT. The initial in vitro experiments on ECT were carried out over 20 years ago 6, and since then many detailed clinical trials, for example, for the treatment of cutaneous and subcutaneous tumors 8 as well as deep‐seated tumors 9, 10, 11 have been reported. There have been numerous publications on the application of ECT in other organs and different animal models as well, such as application for the treatment of cancerous tissue in bone 10, brain 11, and liver 12.

Electroporation by itself can function as an ablation modality, when high enough voltages are applied, which results in an irreversible damage of the membrane, causing the cells to lose their homeostasis and eventually die 13, 14, 15. It is known that during each IRE application, an additional zone of reversible electroporation (RE) occurs automatically in the surrounded area 13. The created bigger penumbra makes it possible to extend the treatment area to lesions that go beyond the lymph node, and can be used to apply ECT. The combination of Irreversible Electroporation and Electrochemotherapy, IRECT, makes it possible to treat large tumor areas without causing damage to local organs or tissue, making it a gentle form of therapy. Critical structures can thus be spared. The drug of choice is usually bleomycin, as previous studies have shown great efficiency after only one application 9. The increased efficacy caused by bleomycin extends to the IRE treatment area margins, in which the reversible pore formation is initiated. The combination has been suggested before 13 and studied in the past for the treatment of glioma 16.

Here we report the safe use of the IRECT procedure to treat a patient with metastases in the lymph nodes that needed a sufficiently large treatment area due to the size and the location of the cancerous tissue.

## Case Presentation

### Clinical Data

We report on a 57‐year‐old female patient, presenting with a history of repeated pain in the upper abdomen, having lost weight of approx. 4 kg within 3 months. Abdominal ultrasound of December 2014 showed pathologic perigastric lymph nodes and suspicious findings in small curvature indicating gastric cancer. Abdominal computed tomography and MRI confirmed these findings. At gastroscopy proliferative, tumor formations with central ulceration were detected at a distance of 45–48 cm from the dental arcade. Histology proved diffuse infiltrating gastric adenocarcinoma. CT and bone scintigraphy did not show peripheral metastasis.

**Figure 1:**
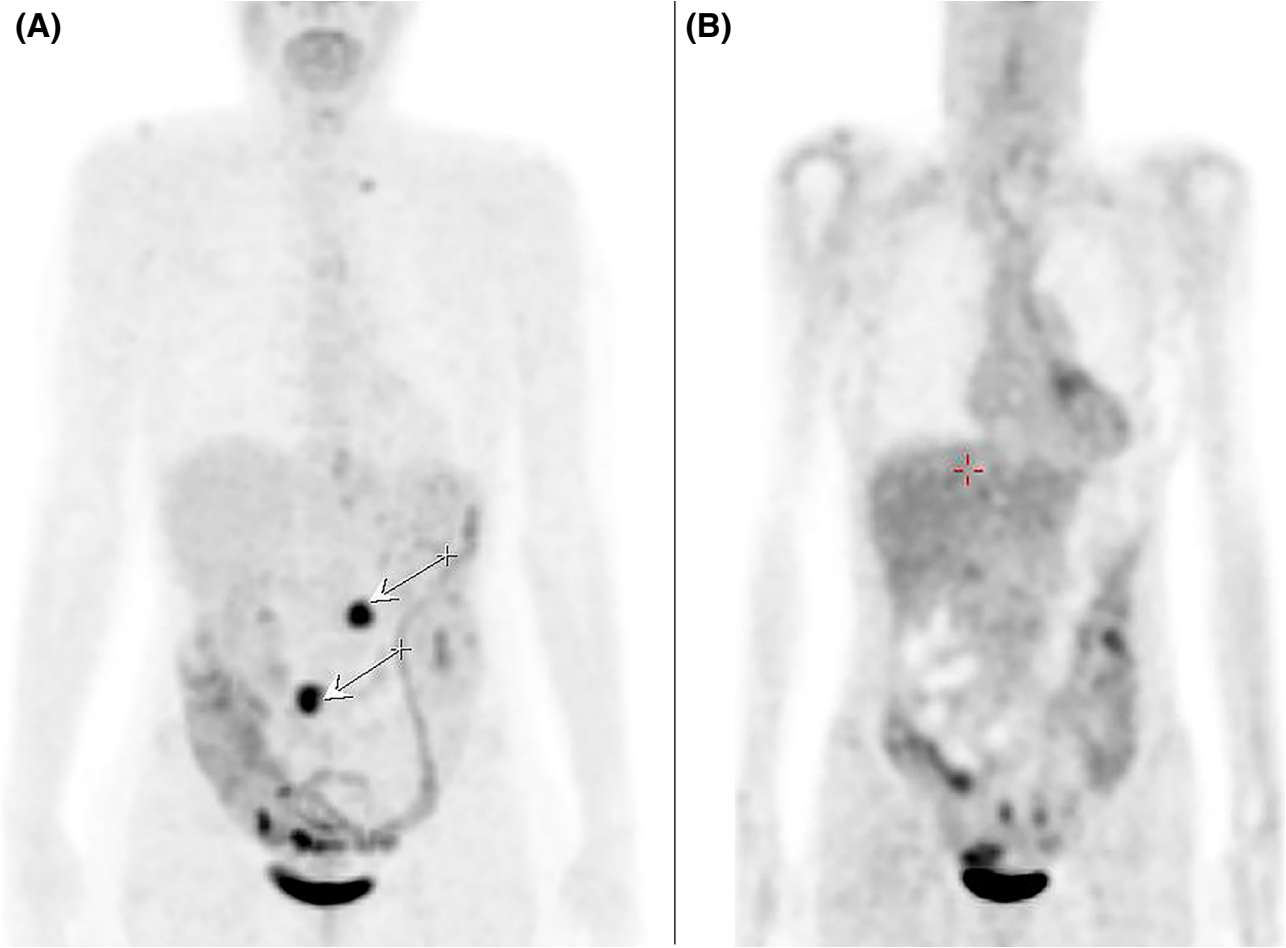
Images show the results of the fluorodeoxyglucose positron emission tomography/computated tomography (FDG‐PET/CT). A. discloses progressive disease with suspicious FDG‐uptake in two lymph nodes (arrows) pre IRECT treatment. B. shows the patient 6 weeks after IRECT treatment.

Blood analysis was unremarkable except for a marked increase in CA19‐9 level to 233 U/mL. The patient underwent neoadjuvant chemotherapy with three cycles of epirubicin, cisplatin, and 5‐fluorouracil (ECF). In May 2015, she underwent total gastrectomy with Roux‐Y‐anastomosis and lymphonodectomy. Pathological assessment revealed poorly differentiated adenocarcinoma of small and greater curvature infiltrating all layers of gastric wall and fundus involvement, four out of 13 lymph nodes affected, one with capsular breakthrough, resulting in pT4aN2. The patient then received two further cycles of ECF, during which follow‐up FDG‐PET/CT showed progressive disease with suspicious FDG uptake in two lymph nodes (Fig. 1A).

**Figure 2:**
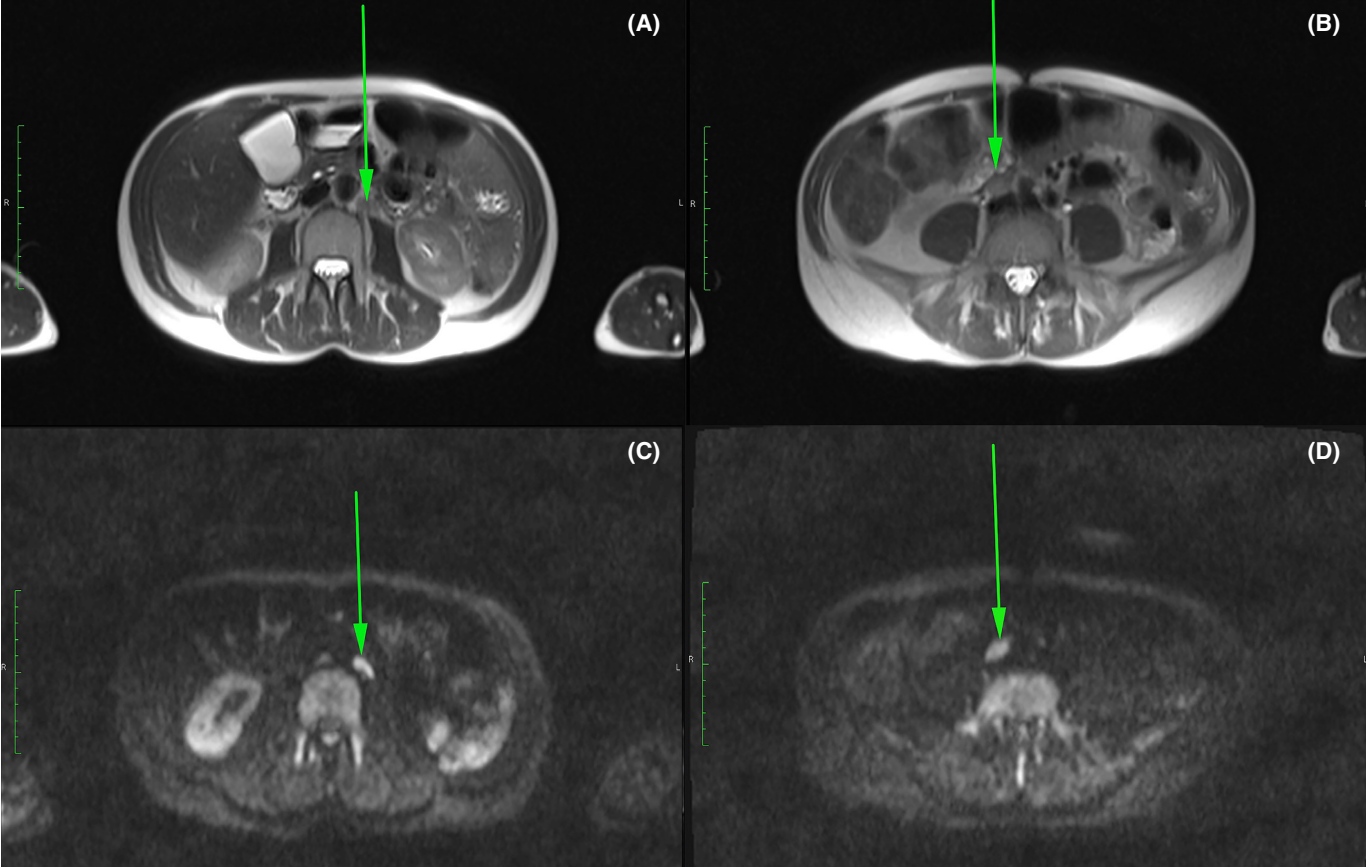
Magnetic Resonance Images (MRI) of the patient pre‐treatment, showing the metastatic lymph nodes in peritoneum and retroperitoneum (arrows) in t2 (A + B) and diffusion‐weighted (C + D) images.

The patient was then referred to our clinic for further treatment. She presented in very good clinical condition without any impairment or complaints (Karnofsky scoring of 100%). Corresponding to PET‐CT, diffusion weighted MRI showed metastatic lymph nodes in peritoneum and retroperitoneum, respectively (Fig. 2). The combination of IRE and ECT was offered as a local palliative treatment option, to which the patient agreed to, and a full informed consent was given.

**Figure 3:**
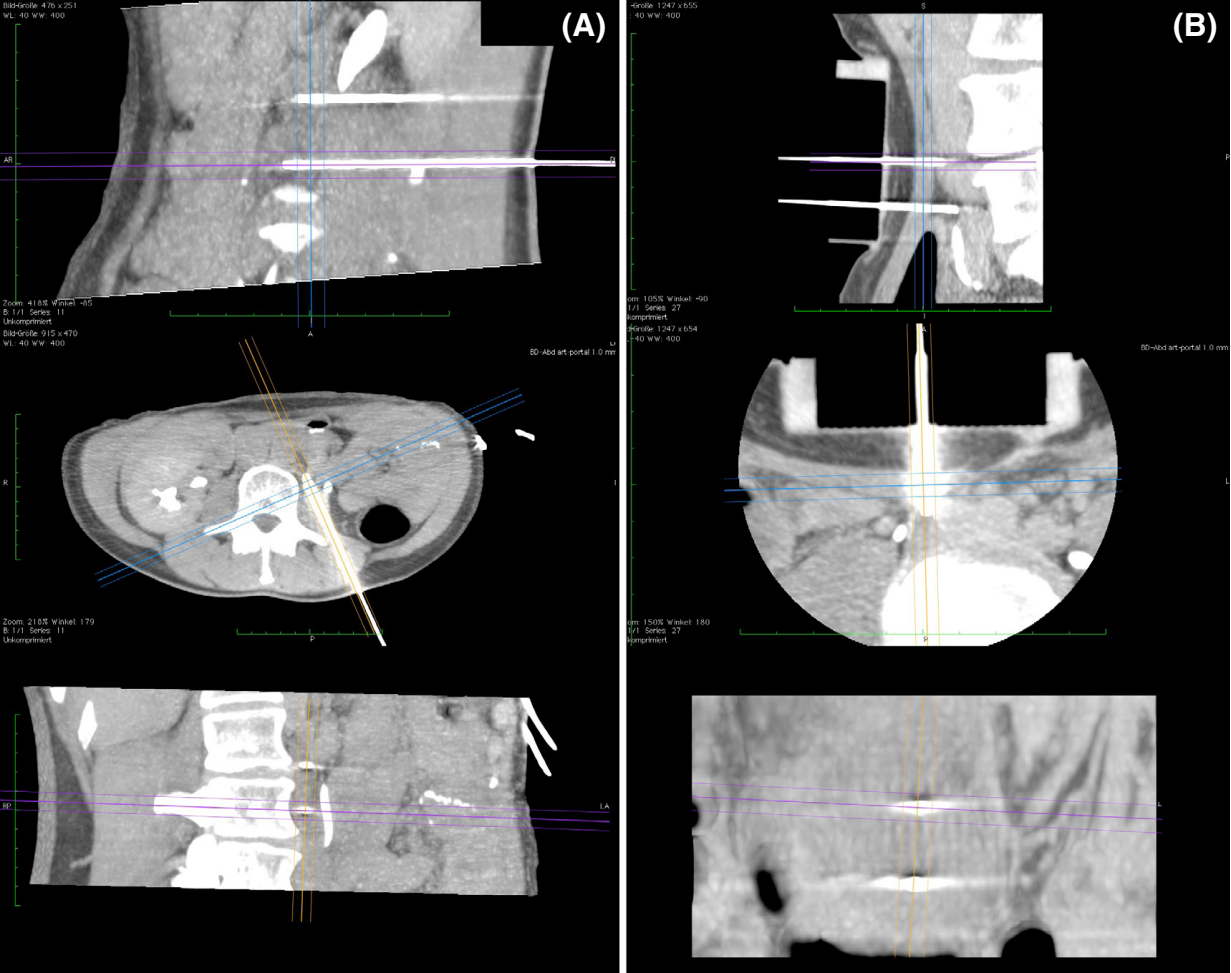
CT images of the patient during the intervention, showing the positioning of the electrodes. A. Posterior approach: Percutaneous insertion of the electrodes through the paraspinal musculature and the psoas muscle for the treatment of a metastatic lymph node located para‐aortal. B. Anterior approach through the lower abdominal wall: Treatment of the second metastatic lymph node, located pre‐caval, with the patient lying in the supine position.

The patient was monitored intraoperatively and 2 h postoperatively on an outpatient basis, a follow‐up MRI was taken after 1 day (Fig. 3A and B), and tumor response control was carried out after 6 weeks by means of FDG‐PET/CT (Fig. 1A) and after 8 months by means of MRI (Fig. 3C and D).

### Intervention

**Figure 4:**
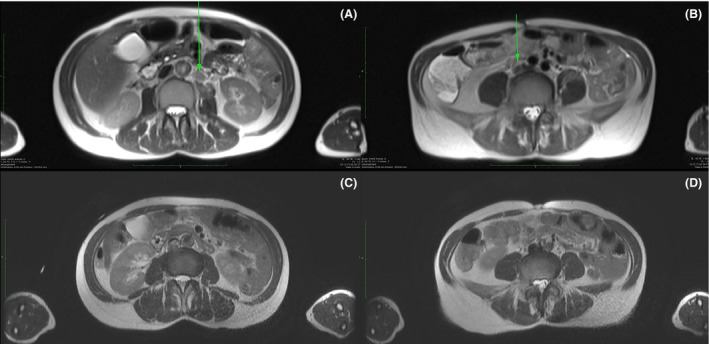
Follow‐up Magnetic Resonance Images (MRI) of the patient after IRECT. A and B. 24 h post IRECT, showing no signs of bleeding, hematoma, fluid or air collections in the two treatment areas. C and D 8 months post IRECT, the treated lymph nodes show no signs of malignancy.

Under total intravenous anesthesia (TIVA), the patient was positioned in the CT scanner (Aquilion / RXL 32‐slice CT scanner, Toshiba) in prone position; CT of retroperitoneum was carried out with additional administration 100 mL of contrast agent i.v (Imeron 300, Bracco IMAGING Deutschland GmbH, Konstanz, Germany). The nondepolarizing muscle relaxant mivacurium (Mivacron^®^, GlaxoSmithKline GmbH & Co. KG, Munich, Germany) was used due to its short half‐life time, avoiding the need for antagonists. A metastatic lymph node in the para‐aortal region was identified. Under sterile conditions, two electrodes were percutaneously positioned under CT and infrared guidance (CAS‐ONE IR, CAScination AG, Bern, Switzerland) at a distance of 21 mm (Fig. 4A) and connected to the generator output of the Cliniporator Vitae (IGEA, Carpi, Italy), which delivers packages of eight 100 ms square pulses with a rise time of 1 msec and at a frequency of 4 Hz.

After the treatment of the first lymph node, the patient was repositioned supine. Focal compression was applied to the central abdomen to displace the bowel loops, and another CT was then carried out to identify the second mesenteric lymph node. Again under sterile conditions, two electrodes were positioned (Fig. 4B) at a distance of 18 mm between one another with guidance of a custom‐made grid of 5‐cm thickness. Bleomycin (Bleomedac, medac GmbH, Wedel, Germany) was administered by intravenous push within 40 sec at a total dosage of 23 mg, 11.5 mg each session, according to ESOPE 9 8 min prior to the delivery of electric pulses. The treatment protocol for the first lymph node was as follows: Eight pulses 2500 V, eight pulses 3000 V, eight pulses 3000 V, and eight pulses 1800 V, eight pulses 2500 V, eight pulses 3000 V for the second lymph node, respectively. The total treatment duration was 3 h 45 min.

## Results and Discussion

Treatment was without any major side effects. Despite muscular relaxation, mild muscular contractions were observed during electrical pulsation, and no changes in cardiologic (ECG, pulse rate) and hemodynamic parameters were noticed. The patient tolerated the treatment very well with only minor complaints (pain intensity according to VAS of about 1–2) that were sufficiently treated with novaminsulfon and diclofenac. Abdominal MRI 24 h after intervention showed mild edema of the psoas muscle in the treated area, while no bleeding, no hematoma, no fluid, or air collections were found (Fig. 3A and B). Patient could be discharged thereafter.

Follow‐up FDG‐PET 6 weeks after intervention was completely uneventful without evidence of any tumor activity (Fig. 1B). No adverse effects concerning intervention or bleomycin toxicity were reported. Follow‐up MRI 8 months after the treatment showed connective tissue conglomerate without malignancy suspicious ADC values in either lymph nodes (Fig. 3 C and D). To this date, the patient reported no complaints.

Gastric cancer is a common form of cancerous disease which is often treated by means of gastrectomy and an additional dissection of the lymph nodes. The most common treatments of lymph node metastases are complete surgical excision, chemotherapy, or radiation therapy. Patients with lymph node metastases who reject these therapeutic options seek to find an alternative therapy. The presented case report illustrates the safe application of IRECT for minimally‐invasive local tumor control, which has several advantages.

IRE will cause permanent damage to the cellular membrane of the cells within the treatment field, while within the area around the IRE field, the reversible version of the same effect occurs, making an increased uptake of antitumor drugs possible. Both the methods separately and the combination of the two are known to be efficient local therapeutic modalities for the palliative treatment of unresectable recurrent tumor nodules 17. In combination, it is possible to extend the treatment area while preserving the gentle quality of both therapies.

No major adverse events from either the chemotherapeutical drug or the pulse deliveries could be reported in this case study. Follow‐up MRI showed no damage to the surrounding tissue or organs (Fig. 3), illustrating the gentle form of this approach. The tolerability is affirmed by the fact that the pain intensity was minimal. Additionally, local treatments such as IRE, ECT, or IRECT allow both several sessions and the combination with other treatment methods, making them adjustable to the patient's needs.

We were able to demonstrate the practicality of the application of IRECT at two different locations in the body and with two different approaches, namely posterior and anterior (Fig. 4). However, it should be noted that the insertion of the electrodes needs to be planned out precisely and with great care to avoid damage of organs and vessels within the insertion pathway.

The lymph node metastases were treated within less than 4 h, and a single dose of bleomycin (a total dosage of 23 mg) was applied, as opposed to several cycles of chemotherapy within a longer period of time, where response rate and benefit of the procedure may be limited 18. It is known that the application of bleomycin in ECT has different response rates, depending on the histology and size of the tumor 19. With IRECT, it may be possible to extend the treatment area for larger tumors, without causing damage to the surrounding tissue. It is therefore necessary to obtain data on response rates for different cancer types and different tumor diameters for IRECT application to further investigate efficacy of IRECT. It should also be noted that we present a follow‐up period of 8 months. To fully investigate the efficacy of IRECT, a clinical trial in a larger patient group with longer follow‐up will be necessary.

## Conclusion

We present the first case of the treatment of lymph node metastases from gastric cancer with a combination of Irreversible Electroporation (IRE) and Electrochemotherapy (ECT), IRECT. Side effects, which were experienced by our patient, were minor and could be treated sufficiently with novaminsulfon and diclofenac. 8 months follow‐up show no evidence of tumor activity, and surrounding organs and tissue were completely spared. Our result shows that the treatment was well tolerated and safe. We therefore recommend the consideration of further clinical studies in a bigger patient cohort to obtain detailed information on tolerability, efficacy and response rate of IRECT in the treatment of deep‐seated tumors and cancerous tissue.

## Authorship

NK, SZ, EG, MKS: all contributed to the preparation, reviewed, and submitted the manuscript.

## Conflict of Interest

The authors report no relevant conflict of interests.
